# Associations and Outcomes Between Chronic Traumatic Encephalopathy and Vasculitis in Adult Patients

**DOI:** 10.7759/cureus.6795

**Published:** 2020-01-28

**Authors:** Dennis Adjepong, Bilal Haider Malik

**Affiliations:** 1 Neurological Surgery, California Institute of Behavioral Neurosciences and Psychology, Fairfield, USA; 2 Internal Medicine, California Institute of Behavioral Neurosciences and Psychology, Fairfield, USA

**Keywords:** chronic traumatic encephalopathy, traumatic brain injury, blood-brain barrier, vasculitis, effects of chronic traumatic encephalopathy

## Abstract

Chronic traumatic encephalopathy (CTE) results from brain injuries and traumas due to accelerated impacts on the head. In severe cases, the diseases cause brain damage, given the head trauma. On the other hand, vasculitis occurs through antibodies that mistake protein vessels as foreign, hence fighting them and resulting in their damage. Examination is usually conducted through blood tests, with antibodies being identified in the antineutrophil cytoplasm. It is unfortunate that its devastating effects also affect the brain of a human, hence leading to dis-functioning. When vasculitis is left untreated, it results in multiple adverse effects on the human body and health both in the short term and in the long term. This study aims to bring to the awareness of neurosurgeons the associations between CTE and vasculitis. This study has proved that there is a close correlation between the progression of CTE and vasculitis. The inflammatory of the blood vessels, as witnessed in vasculitis, increases the risk factors for CTE. The scaling of the vessels and manifestation of different vasculitis conditions in active central nervous system cells results in the worsening of neurodegeneration of the CTE disease.

## Introduction and background

Chronic traumatic encephalopathy (CTE) is a condition that occurs as a result of multiple mild traumatic brain injuries (TBIs) [1-2]. The neurodegenerative disease is known to commonly affect military veterans and athletes who are likely to experience repeated head blows [3]. Vasculitis, on the other hand, occurs as blood vessel inflammation resulting in the weakening, thickening, and stretching of the vessel, which prevents normal blood flow [4]. The reduction of blood flow is among one of the risk factors of vasculitis as it can result in damaging tissues and organs including the peripheral nervous system, central nervous system (CNS), and the brain [5]. The TBI resulting in CTE brings the association between CTE and vasculitis [6]. TBI can result in the formation of hemorrhages interpreted through vascular integrity and degeneration [7]. This process causes rupturing of the small vessels and formation of basilar artery aneurysm on the focal vasculitis [8-9]. The infiltration of the small thalamic vessels highlights the issue of vascular degeneration and trauma-associated vasculitis among CTE patients [10]. This research paper provides an overview of the association between CTE and vasculitis, as well as their pathophysiology such as complications, treatment, and disease progression.

## Review

This paper will use a traditional review to gather information about CTE and vasculitis. The study will take advantage of clinical trials to examine the scientific analysis of the diseases and genetic factors that might contribute to the condition, and identify any gaps in the research [2].

The primary characteristic that links CTE to vasculitis is the inflammation of the blood vessels. Research shows that a combination of these conditions occurs in adult patients, especially military veterans, American footballers, and other athletes who play sports such as hockey and boxing. Research shows that sub-concussive or repetitive concussive blows increase the risk of brain injury trauma, which occurs as a result of the blood-brain barrier and microvasculature.

Pathophysiology of the disease

Mainly, the pathophysiology of CTE is a result of damage to the axons, which occurs due to repetitive traumatic injury. The neurodegeneration of the affected brain tissues is worsened by the glutamate receptors and pro-inflammatory cytokines [1]. The glutamate clearance interference, along with the combination of nitrogen intermediates and reactive oxygen, increases the response to injury. Other conditions such as latent viral infections, environmental toxins, and systemic infections in the brain also worsen the CTE disease [3]. The underlying pathophysiology of vasculitis is classified depending on the type of disorder. The predominant factor is the reduction of sizes for the small, medium, and large blood vessels. The pathogenesis of vasculitis is contributed by the adaptive and innate immune systems [11]. The location of the inflammation results in a variance in the degree of necrosis or the number of layers forming in the blood vessels [12]. Vasculitis condition is characterized by different traits such as the presence of giant cells, fragmentation of small nuclear cells around the vessels, and intimal hypertrophy. The primary characteristic of CTE is the deposition of p-tau protein, which occurs as neurofibrillary tangles (NFTs). CTE shows an association with vasculitis through the cerebral tonsils scarring, a characteristic visible in vasculitis patients [13]. Advanced and intermediate CTE show changes in the reduction of the white and grey atrophy matter, and the brain weight. In vasculitis patients, ANCAs (antineutrophil cytoplasmic antibodies) determine the specific diagnosis of vasculitis [13].

Research by the National Institute of Bioengineering and Biomedical Imaging and the National Institute of Neurological Disorders and Strokes explained that CTE is considered progressive, with the neurodegenerative disease being characterized by increased P-tau brain deposits. However, its pathophysiology is quite unclear, with animal tests proving deposits of pathogenic cis of phosphorylated Thr231-Pro tau protein related to head trauma. P-tau deposits are then consistent with their diagnosis and characteristics of CTE, with much accumulation triggering neurodegenerative cascades that result in cell deaths alongside dysfunctions in brain networks. In other instances, pathology CTE is evident among patients suffering from temporal lobe epilepsy despite an absence of TBI history.

Clinicians also argue that CTE pathological clinical health demands increased safety and sober assessment of scientific insights and data-enhancing evidence-based practices in clinical manifestations. Clinical symptoms of all individuals must be accessed given their implications on individual health. CTE is dangerous as its exacerbating perceptions may result in increased disability of patients, hence the need to understand and solve the issue accordingly.

Genetics of the disease

CTE has been implicated with the TMEM106B genes, which has been proven scientifically to partially explain why some athletes encounter CTE in severity than others despite being exposed to the same level of head trauma [14]. Genetic variation can be used to examine individuals who are at a high risk of developing the CTE pathology. The study of genetics patterns also helps in explaining the underlying disease mechanism, which is an essential biomarker in the identification of appropriate treatment and respective diagnoses [15]. A study at Boston University showed that athletes who had TMEM106B gene in their brain were highly reactive to inflammation. The genetic risk factors of vasculitis depend on the type of condition; for example, in Takayasu arteritis, HLA (human leukocyte antigen) region occurs as the leading risk factor [16].

Scientific analysis

An accurate scientific study of the CTE condition involves the evaluation of the underlying causes, its diagnosis, and examination of the appropriate treatment method. The experimental investigation of this disease shows an existing gap in research and clinical findings due to the lack of a means to diagnosis CTE while people are still alive [17]. However, individuals with vasculitis and at a high risk of CTE due to repetitive head traumas can undergo early treatment to prevent the progression of the disease and premature death [18]. The presence of neurodegenerative tissues is identified using a PET (positron emission tomography), which detects abnormalities in the p-tau.

Clinical analysis of the disease

Mild CTE is associated with depressive symptoms and, short-term memory loss, as well as aggressive traits [17]. In stage 2 of CTE, patients portray explosive behavioral and mood symptoms, which worsens with severe depressive symptoms [19]. In the last two stages of CTE, patients undergo cognitive deficits, psychotic symptoms, language deficits, Parkinsonism, and motor deficits [20]. Vasculitis can manifest autoimmune diseases; it results in an increased risk of atherosclerosis and cardiovascular events, particularly among rheumatoid arthritis patients.

The unanswered questions

The underlying pathogenic mechanism or the events initiating the occurrence of vasculitis remains to be unknown. The research also uncovers that the pathogenic antibodies linked with the inflammation of the large-vessels vasculitis have not been identified [21]. There is also a limited study on the association between CTE and vasculitis [22]. Therefore, questions such as whether vasculitis hinders the treatment of CTE or whether the treatment outcomes are influenced remain to be inadequately addressed. This study has proved that there is a close correlation between the progression of CTE and vasculitis [23]. The inflammatory of the blood vessels, as witnessed in vasculitis, increases the risk factors for CTE [24]. The scaling of the vessels and manifestation of different vasculitis conditions in active CNS cells result in the worsening of neurodegeneration of the CTE disease [25]. The clinical interventions of CTE show the need for avoiding head injury, which might result in the inflammatory [26]. This research has identified a gap in the treatment and diagnosis of CTE as they can only be performed in an autopsy [11]. However, the research was limited to the existing research articles, which made it difficult to obtain accurate data regarding the association between CTE clinical outcomes and vasculitis [12]. Although the primary risk factor for the CTE is repetitive head injuries, the presence of vasculitis can increase the susceptibility of a person to CTE due to high inflammation response [18].

## Conclusions

This research article has shown a strong correlation between CTE and vasculitis. Despite the gaps in our understanding, the primary risk factors of CTE are repeated head injuries, whereas the presence of vasculitis increases inflammation, which worsens a patient condition. There is a need to urge the research community to focus more on these associations and come up with strong statistics between vasculitis and CTE.
